# Prospective Views for Whey Protein and/or Resistance Training Against Age-related Sarcopenia

**DOI:** 10.14336/AD.2018.0325

**Published:** 2019-02-01

**Authors:** Yuxiao Liao, Zhao Peng, Liangkai Chen, Yan Zhang, Qian Cheng, Andreas K. Nüssler, Wei Bao, Liegang Liu, Wei Yang

**Affiliations:** ^1^Department of Nutrition and Food Hygiene, Hubei Key Laboratory of Food Nutrition and Safety, Tongji Medical College, Huazhong University of Science and Technology, Wuhan, China.; ^2^MOE Key Lab of Environment and Health, School of Public Health, Tongji Medical College, Huazhong University of Science and Technology, Wuhan, China.; ^3^Department of Traumatology, BG Trauma center, University of Tübingen, Tübingen, Germany.; ^4^Department of Epidemiology, College of Public Health, University of Iowa, IA 52242, USA.

**Keywords:** age-related sarcopenia, whey protein, resistance training, sex hormones, gender differences, gut microbiota

## Abstract

Skeletal muscle aging is characterized by decline in skeletal muscle mass and function along with growing age, which consequently leads to age-related sarcopenia, if without any preventive timely treatment. Moreover, age-related sarcopenia in elder people would contribute to falls and fractures, disability, poor quality of life, increased use of hospital services and even mortality. Whey protein (WP) and/or resistance training (RT) has shown promise in preventing and treating age-related sarcopenia. It seems that sex hormones could be potential contributors for gender differences in skeletal muscle and age-related sarcopenia. In addition, skeletal muscle and the development of sarcopenia are influenced by gut microbiota, which in turn is affected by WP or RT. Gut microbiota may be a key factor for WP and/or RT against age-related sarcopenia. Therefore, focusing on sex hormones and gut microbiota may do great help for preventing, treating and better understanding age-related sarcopenia.

Aging is the physiological function decline in organs over time eventually leading to death. In 2011, the World Health Organization (WHO) estimated that the number of people aged over 65 would increase from 524 million in 2010 to nearly 1.5 billion in 2050, and likewise, it would grow from 110 million to 330 million in China over the same period. Aging of the population is accompanied by different kinds of age-related complications and deuteropathies, such as Alzheimer’s Disease (AD) [1], Parkinson’s disease [2] and sarcopeniav[3]. Although sarcopenia is also correlated with cachexia induced by cancer [4, 5] or other severe chronic wasting diseases [6, 7], we only focus on the normal age-related sarcopenia in the present review.

Skeletal muscle aging is characterized by decline in skeletal muscle mass and function with growing age, which consequently leads to age-related sarcopenia, if without any preventive timely treatment through appropriate nutrition and exercise. Due to its association with many serious consequences like fractures, falls and disability, age-related sarcopenia results in reduced quality of life and independence [8]. Besides, muscle atrophy may affect several physical functions such as regulation of glucose and production of hormone [9], negatively influences the recovery from diseases in need of large protein reservoir [10] and elevates risk of mortality [11]. It’s been estimated in 2001 that over eighteen billion dollars of healthcare cost were spent additionally on age-related sarcopenia in USA [12]. Many researchers have been looking for the effective prevention for age-related sarcopenia because of its great impact on elder people as well as on social resources. Available evidence suggests that supplementation with proteins, especially whey protein (WP), holds the potential to prevent sarcopenia. Protein intake plays a critical role in muscle mass, strength and functionality in general and especially important for elder people [13]. After a 3-year follow-up observation, the elder, community-dwelling adults with highest quintile of protein intake kept nearly 40% more lean mass and appendicular lean mass than those with the lowest quintile [14]. Beasley et al. demonstrated that higher calibrated protein intake at baseline in postmenopausal women contributes to higher and a slower declining grip strength as well as to more chair stands[15]. Many experts also recommended that a higher protein intake of 1.0-1.2 g/kg b.w. per day should apply to healthy elder people in order to exert positive effects on this population [16-18].

As a matter of fact, different proteins have different compositions of amino acids [19, 20] and varying digestion rates [21]. WP, casein and soy protein are the three main proteins shown to promote muscle protein synthesis (MPS). For instance, both WP and soy protein can induce great MPS response in elder men at rest and after resistance training (RT)[22]. Burd et al. found that WP isolate and micellar casein could support high rate of MPS both at rest and after RT in healthy elderly men [23]. Meanwhile, the two studies also mentioned that WP could promote greater MPS response than the other two proteins [22, 23]. Similarly, other studies have shown that WP is a stronger stimulant for MPS than other proteins [24-28]. Moreover, it seems that WP, also enriched in leucine and combined with RT can support stronger MPS in elderly men compared to the control group [23, 29], implying that RT and leucine may also play a key role in MPS response.

However, previous studies mostly focused on the effect of WP supplementation with or without RT on MPS in elder people and the comparisons between different proteins. In addition to the aspects mentioned above in this review, we are going to discuss gender differences in skeletal muscle and aged-related sarcopenia as well. Besides, plentiful studies revealed that gut microbiota may have influence on MPS and skeletal muscle aging as well [30] (Fig. 1). Hence, there is a need to review available evidences for a better understanding of WP against age-related sarcopenia in elder people.

**Figure 1.**
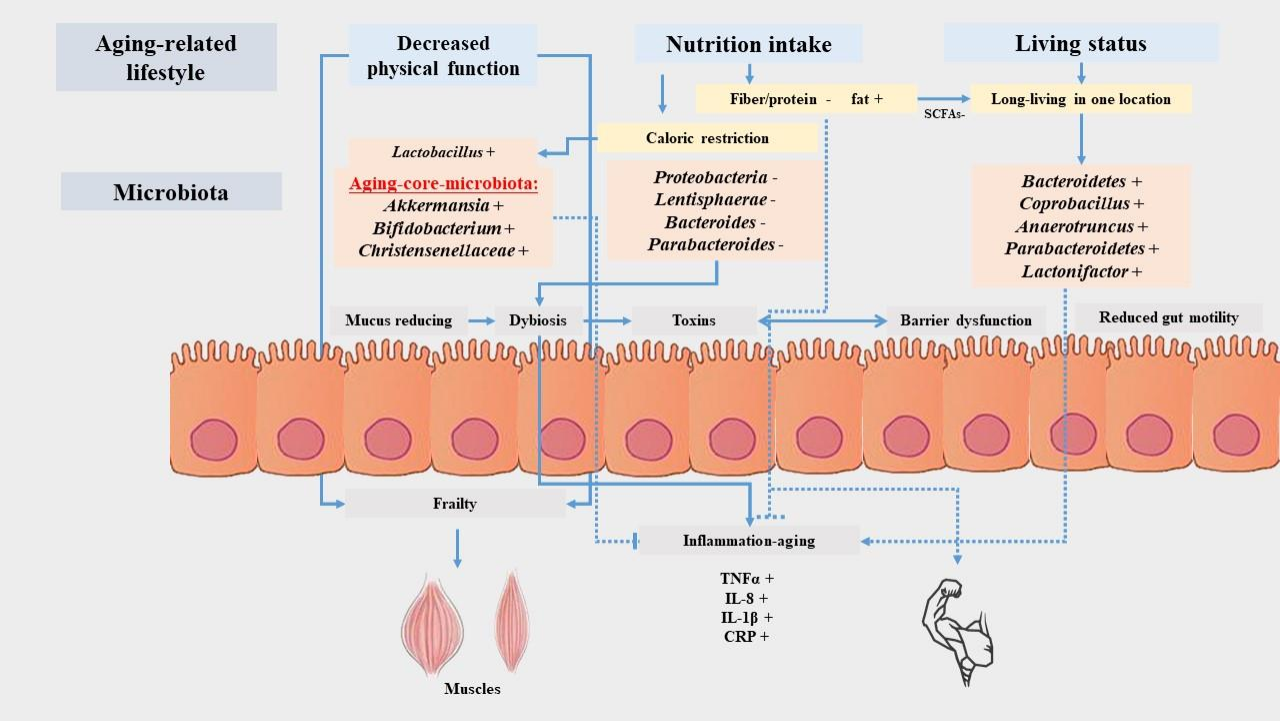
**Gut microbiota and muscle aging**. Age-related lifestyles, including decreased physical function, nutrition intake and living status, would induced the changes of gut microbiota. The increased *Bifidobacteria, Christensenellaceae*, and *Akkermansia* were identified as aging-core-microbiota. The substantial microbiome change in aging may affect changes in gut physiology such as reduced gut motility, reduced mucus, barrier dysfunction, and dysbiosis, which can further mediate the translocation of bacterial toxins and muscle aging. “+” means increased, “-” means decreased.

## Skeletal muscle aging and age-related sarcopenia

There are more than 600 skeletal muscles in human body, which comprise nearly 40% of total body mass. Cooperating with bones and joints, skeletal muscles help human and other mammals with their various body movements via contraction and relaxation. After the age of 50, muscle mass declines at a rate of 1-2% per year on the average [31, 32]. Kyle et al. also illustrated that the decline in fat-free mass and appendicular skeletal muscle mass is accelerated in men and women after 60 [33]. Muscle mass and function begin to decline at the same time [34], but muscle function declines more quickly than muscle mass [35], indicating that function may be more sensitive to aging [36]. Meantime, a 12-year longitudinal study revealed that quantitative loss in muscle cross-sectional area is a major contributor to the decrease in muscle strength [34]. 5%-13% of elder people aged 60-80 and 11%-50% of those aged over 80 are suffering from severe muscle loss [37, 38], which demonstrates that the prevalence of sarcopenia increased along with the growing age.

There is still lack of a general international consensus on the definition of sarcopenia, although Edwards et al. enumerated four definitions of sarcopenia emphasizing the importance of definition for diagnosis of this condition and pointing out the difficulties to define it [36]. At present, relative skeletal muscle index (RSMI), appendicular mass relative to height squared, is mainly used as a parameter to diagnose sarcopenia clinically [39], with RSMI < 7.23 kg/m^2^ for men and < 5.67 kg/m^2^ for women. Other parameters, such as body mass index (BMI), grip strength and muscle strength, are also applied as auxiliary means to diagnose sarcopenia [40].

An outstanding study involving model organisms indicates that both muscle tissue external and internal factors contribute to the appearance of sarcopenia[41]. Lower nutrient intake, deficient physical activity and hormone levels in elder people affect the regeneration of skeletal muscle tissue, and so does the neuronal loss, like fiber denervation, and declined regenerative ability, like the dysfunction of satellite stem cells [41]. Besides, sarcopenia would augment susceptibility to many diseases such as cardiac failure and spinal injury, whereas the drugs for these diseases often result in muscle weakness to promote sarcopenia in turn [42, 43]. On the other hand, the primary internal causes of sarcopenia are organelle dysfunction and compromised protein homeostasis, such as increased mitochondrial dysfunction, increased DNA damage, increased fiber atrophy, increased protein modification and damage, accumulation of damaged proteins and organelles, defects in the function of contractile proteins and sarcomeres and so on[41]. Among these complicated factors, the declining mitochondrial function in skeletal muscle has been widely investigated as a significant way to induce sarcopenia [44, 45].

Age-related sarcopenia is a risk factor for falls and fractures, leading to disability, dependency, poor quality of life, increased use of hospital services and mortality [46]. A 2-years follow-up illustrated that people aged 80 years or older with sarcopenia are over three times more prone to fall relative to non-sarcopenic individuals, regardless of age, gender and other confounding factors [47]. Low thigh muscle cross-sectional area augmented the risk of incident mobility limitations and fractures in community-dwelling older people [48, 49]. Other studies also demonstrated that disability [50, 51], cimpairment in activities of daily living [52] and mortality [53] are severe consequences of sarcopenia. Therefore, it’s urgent to find an effective solution for sarcopenia due to its serious consequences mentioned above.

## Whey protein and/or resistance training against age-related sarcopenia

### Whey protein promotes muscle health in human and animals

All of the macronutrients can be used for energy production in human body, but only protein also serves as a structural and functional compound in all organs and cells [54]. Among all kinds of proteins, WP was initially regarded as a useless byproduct of cheese production for decades [55], but turned into an extremely popular nutritional supplement afterwards owing to its high digestibility, quick absorption and rich content of essential amino acids (EAAs) [56]. A double-blind crossover study demonstrated that WP supplementation can enhance whole body protein anabolism [57], which is considered as the most remarkable and fundamental effect on human body. Besides, WP showed promise in the prevention and treatment of obesity and type 2 diabetes mellitus in both human and laboratorial animals [58-61]. In addition, several studies have shown that WP also plays a positive role in blood pressure control [62], anti-inflammation [63] and anti-oxidative stress [64-66].

An increasing number of studies has illustrated that WP has a positive effect on MPS in both healthy young and elder people [23, 24, 28, 67-69]. For instance, a randomized controlled trial (RCT) showed that WP stimulates gains in lean body mass and strength in healthy elder men [70]; likewise, WP excites MPS response in healthy elder women[71, 72]. However, another study suggested that WP supplementation for two years could not promote MPS to enhance muscle mass and physical function in healthy elder postmenopausal women [73]. Tang et al. also found that WP hydrolysate is a great stimulant for MPS in healthy young men [69]. Moreover, MPS induced by WP was also observed in rats [74, 75]. However, Mosoni et al. reported that although high WP intake delays the loss of lean body mass in healthy old rats, it is correlated with a reduction in muscle proteolysis rather than mediated by MPS [27].

Leucine is a high proportion of the branched-chain amino acid (AA) in WP [76], which was reported to be one of the crucial factors for the stimulation of MPS according to many researches [29, 77-79]. A RCT designed by Luiking et al. demonstrated that leucine-enriched WP leads to a larger overall postprandial MPS rate in healthy elder men and women than a conventional dairy product [29]. AA ingestion improves MPS in the young and elderly [80]. Whereas high proportion of leucine can reverse an attenuated response of MPS in elderly, it cannot further stimulate MPS in young subjects [77]. Moreover, leucine-rich WP can improve postprandial muscle protein synthesis in aging rats [79]. In addition, cysteine, another AA in WP, was illustrated to be another key element to influence muscle mass and function [81-83].

The above discussion was mainly about the effect of WP on MPS of healthy elder people. The question is, if there is the same effect on MPS can be observed in unhealthy elder people as well? A study on 380 sarcopenic older adults revealed that sufficient baseline levels of vitamin D and leucine-enriched WP are conducive to gaining more appendicular muscle mass[84], which is consistent with another study on 130 sarcopenic elderly people [85]. Hector et al. illustrated that WP can retard the declining rate of MPS during the short-term weight loss interventions in overweight and obese men and women aged 35-65 years [86]. Similarly, a double-blind RCT on 80 obese older adults confirmed that high WP-, leucine-, and vitamin D-enriched supplement compared with an isocaloric control preserves appendicular muscle mass during a hypocaloric diet and RT program. The general improvement in muscle strength and function could be observed, however without any significant differences between groups [87]. Also, WP ingested during the exercise can lead to greater whole-body protein synthesis in patients with chronic obstructive pulmonary disease (COPD) compared with the healthy control group[88]. However, there are no significant differences in effects of leucine-enriched WP and commercial milk product on muscle mass and functions in elder people suffering from polymyalgia rheumatica [89]. What’s more, animal studies demonstrate that nutritional intervention with antioxidants and/or leucine-enriched WP can play an important role in improving muscle function and quality in antioxidant deficient aged mice as well [90].

In summary, WP supplementation indeed promotes muscle health in both human and animals, suggesting that WP ingestion could be an effective means to prevent and counteract sarcopenia in healthy or unhealthy elder people. Meanwhile, the level of leucine should be emphasized when supplementing WP owing to its pivotal role in supporting MPS.

### Resistance training with or without whey protein against age-related sarcopenia

RT is a special physical exercise causing the muscles to contract against an external resistance to build the strength, anaerobic endurance and mass of skeletal muscles. Dumbbells, rubber exercise tubing, one’s own body weight, bricks or any other objects that causes the muscles to contract can be used as external resistance [91]. RT has been proved to be beneficial for humans, including delaying aging [92], reducing fat mass and changing body shape [93], alleviating injury and pain in muscle [94], burning calories [95], preventing and treating metabolic syndrome [96] as well as bone-related diseases [97]. The present review will focus on its function of building muscle strength and mass to prevent and manage sarcopenia in elder people.

Previous studies have shown that RT affects the mass and function of skeletal muscles in elder people [98-100]. Bemben et al. demonstrated on 42 males aged 48-72 years that 3 days for 14 weeks of RT significantly increased muscle strength and mass [101]. Likewise, high volume (intensity) RT (2 days/week for 10 weeks) is effective for improving some indices of muscle mass and strength in postmenopausal women [72]. Step-reduction, a common change in older adults, would cause muscle atrophy and decline in postprandial MPS, but low-load RT can attenuate the deleterious effects of step-reduction in aging muscle [102]. In particular, low-load and high-load RT can alternate in training approaches, since there is no difference in detraining’s effect on muscle volume, muscle strength and functional capacity in older adults in regard of training load could be observed [103]. In addition, RT can impact both fiber cross-sectional area and muscle function[104] while endurance training cannot influence the muscle fiber cross-sectional area [105].

Some studies investigated the joint effects of WP and RT on muscle health, showing a more profound effect from the combined intervention than from WP or RT alone. A meta-analysis of 22 RCTs illustrated that protein (WP, casein, EAA) supplement combined with prolonged RT (> 6 weeks) led to greater gains in lean body mass and strength in both younger and older adults [106]. Another systematic review and meta-analysis showed that compared with RT alone, protein supplement combined with RT may have a stronger effect in preventing aging-related muscle mass attenuation and leg strength loss in overweight and obese older people [107]. The ingestion of WP isolate after unilateral leg RT stimulated stronger MPS in elderly men than ingestion in rest condition [23]. Yang et al. showed that the combined effect of WP ingestion after a bout of unilateral leg-based RT increased rates of MPS in older men to a greater extent than the feeding alone for all protein doses [108]. At the same time, Denysschen et al. and Weisgarber et al. suggested that compared to RT alone, a combined intervention with WP and RT had no additional benefits for promoting muscle health in hyperlipidemic men or postmenopausal women [72, 109].

Collectively, long-term RT should be encouraged to prevent the loss of muscle mass and function in older individuals regardless of the training load. However, WP in combination with RT is likely to be more conducive to advancing muscle health than each of them alone, providing us a more effective approach in prevention and treatment of sarcopenia in older people.

**Figure 2.**
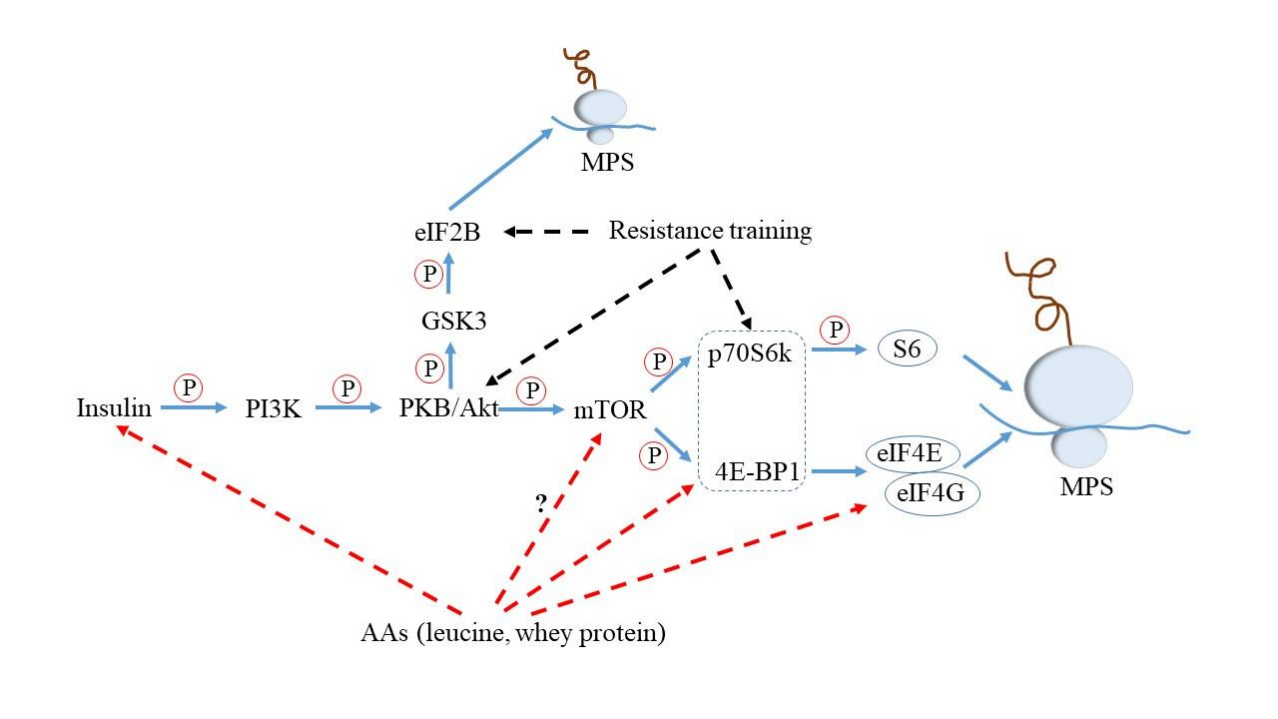
**Mechanism of whey protein/resistance training to induce muscle protein synthesis**. Amino acids (AAs)/resistance training, together with insulin, promote the muscle protein synthesis (MPS) by affecting the components of the phosphatidylinositol 3-kinase (PI3K)-mammalian target of rapamycin (mTOR) signaling pathway, as described in detail in the text. “?”: whether or not AAs directly promote activation of mTOR remains unknown; ?: phosphorylate; PKB: protein kinase B; GSK3: glycogen synthase kinase 3; 4E-BP1: eIF4E-binding protein 1; p70S6k: 70 kDa ribosomal protein S6 protein kinase; elF2B/4E/4G: eukaryotic initiation factor 2B/4E/4G.

### Mechanism of whey protein/resistance training to induce muscle protein synthesis

Up to now, it is widely accepted that the phosphatidylinositol 3-kinase (PI3K)-mammalian target of rapamycin (mTOR) signaling pathway, serving to regulate mRNA translation initiation, is the key mechanism to induce MPS by insulin/AAs (especially leucine)/RT in both human and animals [110-112] (Fig. 2). And studies have shown that insulin and either AAs or RT may act synergistically to stimulate translation initiation and protein synthesis [113, 114].

Insulin would initiate PI3K to regulate mTOR through activation of the insulin receptor’s intrinsic tyrosine protein kinase activity [111]. And protein kinase B (PKB, also known as Akt) acts as an intermediate to phosphorylate mTOR and glycogen synthase kinase (GSK)-3 in the pathway. The mTOR signaling pathway arouses the phosphorylation of its downstream targets 70-kD S6 protein kinase (p70S6K) and eukaryotic initiation factor 4E binding protein 1 (4E-BP1) [115]. Subsequently, p70S6K phosphorylates ribosomal protein S6 to selectively translate 5’-terminal polypyrimidine mRNA encoding ribosomal proteins and translation factors [116], while phosphorylating 4E-BP1 would enable dissociation from eukaryotic initiation factor 4E (eIF4E). Once liberated, eIF4E would bind to eIF4G to promote the uncoiling and binding of mRNA to the 43S ribosomal complex, thereby elevating the translational efficiency [115]. In addition, the phosphorylation of eIF2B by GSK-3 also results in the increased MPS [117].

As we discussed above, there are rich content of EAAs, especially high proportion of leucine, in WP. It was well-known that AAs (especially leucine) enhance phosphorylation of 4E-BP1 and S6K1 [118, 119], two downstream proteins of mTOR, but whether or not AAs directly promote activation of mTOR remains unknown [114]. AAs also stimulate the MPS through the enhanced association of eukaryotic initiation factor eIF4G with eIF4E. Moreover, ingestion of leucine would also trigger a rise in the circulating insulin concentration [120]. Likewise, according to previous studies, RT may lead to increased phosphorylation of Akt/PKB and proteins downstream of mTOR, such as 4E-BP1 and S6K1 [121, 122]. Kimball et al. suggested that RT may stimulate the guanine nucleotide exchange activity of eIF2B in extracts of gastrocnemius muscle to induce MPS as well [114].

Taken together, insulin, acting as the first step to arouse PI3K, can not only play an essential part in regulating mTOR, but also promote great MPS together with leucine. Besides, mTOR is pivotal in the mechanism of WP/RT-induced MPS, indeed.

### Whey protein versus other proteins or amino acids

Although numerous studies have shown the benefits of WP against age-related sarcopenia, other proteins, such as casein and soy protein as well as some AA showed similar effects on muscle health. WP (20%) and different forms of casein (80%) make up protein content in normal bovine milk [55]. Phillips et al. concluded that milk-based protein is better in support of MPS and muscle protein accretion in young and elderly persons than soy-based one [28]. The distinct MPS responses to different proteins are affected to a large extent by the quality of protein [24], depending on different AA content, digestibility and bioavailability. WP, casein and soy protein are complete proteins, which contain an adequate and appropriate proportion of all kinds of the EAA necessary for the dietary needs of humans or animals. Basing on their molecular structure and digestibility whey and soy proteins are regarded as “fast” and casein as a “slow” protein [123, 124].

A number of previous studies have compared WP, casein and soy protein regarding their impact on muscle health. For example, Burd et al. suggested that WP isolate supports greater rates of MPS in healthy older men than micellar casein, both at rest and after RT [23]. Moreover, muscle protein fractional synthesis rate (FSR) was 0.12%/h in the early period (1-3.5 h) of WP ingestion and 0.10%/h in both early and late period (3.5-6 h) of casein intake immediately after RT in young males, suggesting that WP seems to elicit a high but transient increase in MPS [125]. However, by elderly individuals Dideriksen et al. observed no difference in MPS, induced by WP and caseinate supplementation immediately after heavy RT [126]. Soy protein was demonstrated to have a lower ability to induce MPS in elderly men at rest and after RT compared with WP isolate [22]. Whereas, it seems that soy protein doesn’t differ from WP in improving muscle strength after 12 weeks RT in hyperlipidemic males [109]. The effects of mixed WP, casein and soy protein on MPS were investigated as well [69, 74, 75]. It was concluded that according to the their rate in the stimulation of MPS in young men after RT the proteins could be arranged in following order: whey hydrolysate > soy protein > casein [69]. In an animal study, Butteiger et al. suggested that compared to WP alone, a blend consisting of ratio of 25:50:25 for whey: caseinate: soy had a significantly higher FSR at initial peak time of 135 minutes in rats [74]. Kanda et al. demonstrated that the initial peak time in MPS in rats reached by WP first, followed by milk protein and caseinate [75].

In addition, EAAs are recognized as stimuli for muscle protein anabolism as well. WP hydrolysate results in a greater increase in MPS in elderly persons as well as in male Sprague-Dawley rats compared to its constituent EAAs content [25, 127]. However, Paddon-Jones et al. showed that isocaloric ingestion of EAAs might provide a stronger stimulation for MPS in elderly individuals than that of WP [128].

Taken together, we can draw a conclusion that WP is more beneficial than other proteins for muscle health in elder people. It is therefore reasonable to choose WP first when we need protein supplementation for promoting muscle health.

## Gender differences in skeletal muscle and age-related sarcopenia

In general, there are more body fat and less muscle mass in women than in age-matched men, at any given total body weight [129, 130]. Muscle mass in men is on average 36% higher than in women and gender differences are greater in the upper body (40%) than lower body (33%) [131]. These differences of body composition mentioned above are distinct from the infancy to old age, but become most obvious after puberty [132], which definitely causes the differences of losing muscle mass and suffering from age-related sarcopenia between men and women.

The rate of losing fat-free mass and appendicular skeletal muscle mass in men (1.5 kg/decade, 1.0 kg/decade) is faster than in women (0.8 kg/decade, 0.4 kg/decade) both aged over 60 [33]. Landi et al. demonstrated that the prevalence of sarcopenia in male residents (68%) of nursing homes was higher than in female residents (21%), both groups aged over 70 [133]. Another study also by Landi et al. illustrated that sarcopenia is highly prevalent among elderly persons aged over 80 (25%) without gender differences [47]. On the contrary, the prevalence of sarcopenia among the men and women (mean age 67 years) in the Hertfordshire Cohort Study was 4.6% and 7.9% respectively [134], which is consistent with another study with 11.8% in men and 14.9% in women (mean age 73.5 years) [135]. These different and conflicting data makes it difficult to provide the exact numbers on prevalence rates of sarcopenia in elder people. Still, a population-based prospective cohort study showed that the association between muscle strength and mortality was stronger in women than in men [136], implying that women with lower muscle strength are more prone to resulting in death.

Sex hormones in males and females are proposed to be the crux of gender differences in skeletal muscle and sarcopenia. Estrogen has a marked impact on myofiber growth and regeneration as well as on extracellular matrix remodeling in rats during recovery from disuse atrophy [137]. Enns and Tiidus concluded that estrogen can not only affect muscle contractile properties and attenuate indices of post-exercise muscle damage, but also delay the infiltration of inflammatory cells into skeletal muscle after exercise or injury in animals and human[138]. Testosterone administration for 10 weeks in orchidectomized male mice could maintain skeletal muscle mass and strength and enhance resistance to fatigue of muscle [139]. Serra et al. suggested that testosterone increased satellite cell activation and proliferation for muscle regeneration in both young (2-month-old) and aged (24-month-old) male mice [140]. An early study demonstrated that a pharmacological dose of testosterone enanthate (3 mg/kg/week for 12 weeks) increased muscle mass via elevating MPS in normal male subjects [141]. It seems that sex hormones deficiency would contribute to the development of sarcopenia in males and females later in life. All these relevant studies suggest that sex hormones could be a potential contributor to muscle health.

## Gut microbiota: a key factor for whey protein and/or resistance training against age-related sarcopenia?

Intestinal bacterial colonization starts in fetus, but gut microbiota is established after delivery [142]. A healthy gut microbiota usually remains stable and plays a very remarkable role in protecting its host [30] through detoxifying the toxic substances [143], confronting foreign pathogens [144], strengthening immune system [145] and so on. It has been reported that plenty of changes in gut microbiota take place in elder people aged over 65, such as changes in its diversity, composition and functional features [146-149]. Claesson et al. concluded that compared to younger subjects aged 28-46, the intestinal microbiota of the elderly aged over 65 shows temporal stability over limited time but is characterized by unusual phylum proportions and extreme variability as well [150]. In addition, a recent study also showed the significance of microbial genetic variations in modulating host longevity [151]. Thus, it seems that the variations of gut microbiota would lead to physical function changes in elder people due to microbiota’s significance in protecting the host. Moreover, gut microbiota shares some potential connections with WP and exercise on muscle health (discussed in the following paragraphs), which renders it to be a crucial factor against sarcopenia in elder people.

The effect of protein or WP on gut microbiota has been shown in many animal models. A study on dogs suggested that diets rich in protein (crude protein 303g/kg or 304g/kg) increased pH values in colon to decrease counts of *Lactobacilli* and *Enterococci* [152]. Another study on adult rats revealed that high-protein diet (45% protein) for 6 weeks elevated counts of *Escherichia coli* while reduced *Akkermansia muciniphila*, *Bifidobacterium*, *Prevotella*, *Ruminococcus bromii* and *Roseburia* or *Eubacterium rectale*, compared to the normal-protein diet, which causes the alterations of SCFAs (acetate, propionate, and butyrate) and a lower microbial metabolic activity [153]. In addition, protein quality and structure of protein emulsion affect gut microbiota in mice[154, 155]. McAllan et al. also illustrated that WP isolate influenced the populations of *Lactobacillaceae*, *Clostridiaceae*, *Bifidobacteriaceae* at the family level and their corresponding genera (*Lactobacillus*, *Clostridium* and *Bifidobacterium* respectively) as well as proportions of *Rikenella*, *Peptostreptococcus*, *Desulfovibrio* and *Mucisprillum* at the genus level in mice with high fat diet [155], which is consistent with another similar study[156]. Nilaweera et al. presented that the changes of composition in gut microbiota served as a possible approach for WP isolate to promote energy absorption in C57BL/6 mice [157]. Cheese WP can also protect rats against mild dextran sulfate sodium-induced colitis through the stimulation of intestinal mucin synthesis and modification of microflora composition[158]. Particularly, the digestive process of WP contributes to forming potent antimicrobial whey-derived peptides, such as pepsin catalysed lactoferrin to lactoferricin [159]. In brief, protein or WP influences gut microbiota via altering its composition and metabolic activities.

Exercise regardless of training type (acute exercise, chronic exercise, cardio and RT, voluntarily) and load (low, moderate and high) can have a significant effect on human and animal health by influencing gut microbiota [160-162]. On the one hand, exercise may change the bile acids profile [163]. An increase in *Firmicutes* phylum (mainly *Clostridia* class) and decrease in the *Bacteroidetes* phylum were observed in mice with cholic acid[164]. On the other hand, the increased short-chain fatty acids induced by physical exercise are associated with microbiota changes as well [160]. Voluntary wheel running by obesity-prone rats increased the relative abundance of fecal *Streptococcaceae* and decreased one undefined genus in *Rikenellacea* more robustly compared to sedentary weight-matched obesity-prone rats [165]. In addition to influencing gut microbiota directly, voluntary exercise for 5 weeks in mice can diminish polychlorinated biphenyls-induced alterations of the gut microbiota as well [166]. Professional rugby athletes with extreme exercise, involving RT, had a higher diversity of gut microbiota (representing 22 distinct phyla) than the controls [167]. Physical exercise can also modulate gut microbiota in chronic kidney disease patients [168]. Based on the listed evidences, exercise can evoke alterations of gut microbiota in both healthy and unhealthy human and animals.

It has been proposed that gut microbiota is a source of pathogen-associated molecular patterns, such as flagellin and peptidoglycan, that affects amino acid (AA) bioavailability, modulates the production of pro-inflammatory cytokines and produces various metabolites, like bile acids, which are conducive to potentially influence muscle physiology [169]. And Cerda et al. proposed that the lack of gut microbiota would lead to the increasing levels of 5’adenosine monophosphate-activated protein kinase (p-AMPK) and fasting-induced adipose factor (FIAF), which associated with fatty acid oxidation and glucose uptake in skeletal muscle [160]. For example, fiber characteristics and lipid metabolic profiles of skeletal muscle from pigs can be transferred to germ-free mice by gut microbiota, which influences the skeletal muscle development and the lipid metabolic profiles in recipient mice[170]. Bindels et al. illustrated that restoring the *Lactobacilli* levels by adding specific strains could decrease the inflammation and muscle atrophy markers in female BALB/c mice with acute leukemia [171]. Moreover, Steves et al. concluded that alterations in gut microbiota can promote inflammation and change immune response and host metabolism, which in turn may modulate the development of musculoskeletal problems (such as sarcopenia, osteoarthritis and rheumatoid arthritis) and frailty [172]. In addition, Siddharth et al. suggested that gut microbiota may underlie the sarcopenic phenotype of the aged rats through vitamin synthesis, altered lipid metabolism and regulation of growth and immune-related factors [173]. In short, gut microbiota does exert an influence on skeletal muscle by itself or microbiota-dependent metabolites.

**Figure 3.**
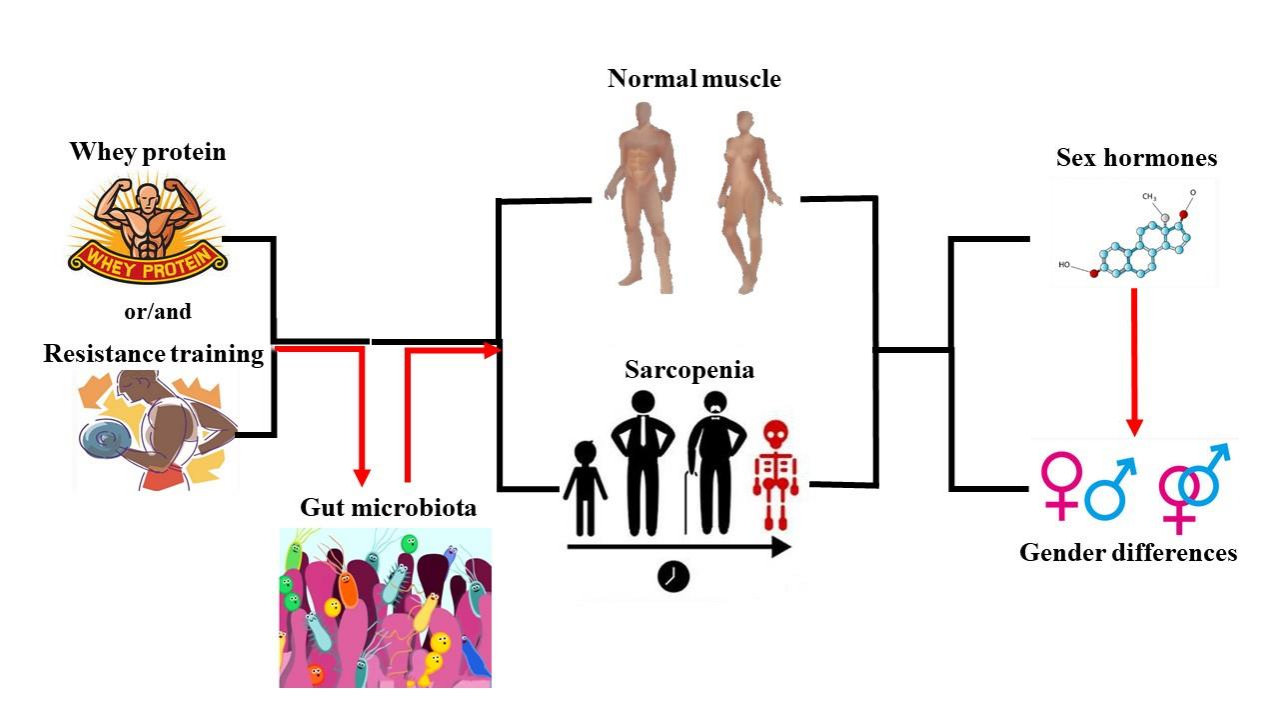
**Schematic diagram of paper structure**. Whey protein/resistance training indeed contributed to age-related sarcopenia in elder people. Besides, it seems that sex hormones could be a potential contributor to muscle health based on the discussion of gender differences in skeletal muscle and sarcopenia. Furthermore, we proposed that gut microbiota may be a key factor in the combination of whey protein and resistance training against age-related sarcopenia.

**Table 1:** Whey protein and/or resistance training promote muscle health.

References	Subjects	Protocol	Outcome
Human (WP combined with RT)			
Burd et al. [23]	14 healthy elderly men (72 y)	20g WP isolate or 20g micellar CA immediately after unilateral leg RT	greater MPS in WP isolate than micellar CA both at rest and after RT
Mitchell et al. [26] D’Souza et al. [119]	13 healthy older men (60-75 y) 46 healthy older men (69.0 ± 0.6 y)	30g WP, 30g SP or a noncaloric placebo immediately after a single bout of unaccustomed lower body RT	phosphorylation of S6K1↑ in SP only at 2h post exercise but in WP at 2h and 4h post exercise
Luiking et al. [29]	20 healthy older persons (> 60 y)	20g leucine-enriched WP (3g leucine) or 6g iso-caloric milk protein immediately after unilateral RT	greater postprandial MPS rate in leucine-enriched WP than milk protein after unilateral RT.
West et al. [57]	12 young trained men (24.0 ± 4.0 y)	25g WP or iso-caloric placebo at 0h and 10h after an acute bout of RT	whole body net protein balance↑ in WP after RT over 10h and 24h compared to the placebo
Tang et al. [69]	18 healthy young men (22.8 ± 3.9 y)	21.4g WP, 21.9g CA, or 22.2g SP at rest and immediately after a bout of unilateral leg RT	greater MPS in WP or SP than CA both at rest and after RT; greater MPS in WP than SP after RT
Bell et al. [70]	49 healthy older men (73.0 ± 1.0 y)	WP-based supplement or a control drink twice daily for 20 weeks (Phase 1); twice weekly RT and once weekly HIIT for 12 weeks after 6 weeks of Phase 1.	MS↑ and lean mass↑ in WP-based supplement; greater MS after RT
Bukhari et al. [71]	16 postmenopausal women (66.0 ± 3.0 y)	20g WP or 3g leucine-enriched EAA immediately after a bout of unilateral RT	Equivalent muscle anabolism in WP and leucine-rich EAA at rest and after exercise
Weisgarber et al. [72]	12 postmenopausal women (57.0 ± 4.7 y)	WP ((4 × 10g aliquots)) or placebo (maltodextrin) during unilateral RT twice weekly for 10 weeks.	muscle mass↑ and strength↑ after high volume RT; WP during RT did not augment this response
Karelis et al. [81]	99 healthy elderly subjects (65-88 y)	20 g/day cysteine-rich WP isolate or CA for 135 days, with RT 3 times weekly	greater MS in WP isolate than CA after RT
Farnfield et al. [112]	16 healthy young (18-25 y) and 15 healthy older men (60-75 y)	WP isolate or placebo drink after each session of RT for 12 weeks	protein phosphorylation↑ in WP isolate with RT; WP- and RT-induced protein phosphorylation↓ in older men, but not in younger men
D’Souza et al. [119]	46 healthy older men (69.0 ± 0.6 y)	10, 20, 30, or 40g of WP or a noncaloric placebo beverage immediately after a single bout of unaccustomed lower body RT	muscle BCAAs↑ during post exercise recovery and larger doses (30 g and 40 g) of WP
Rondanelli et al. [85]	130 sarcopenic elderly people (80.3 y)	A supplement containing 22g WP, 10.9g EAA (4 g leucine), and vitamin D [2.5 mg (100 IU)] with RT for 12 weeks	FFM↑, relative skeletal muscle mass↑, android distribution of fat↑, and handgrip strength↑ after supplement plus RT
Verreijen et al. [87]	80 obese older adults (63.0 ± 5.6 y)	high whey protein-, leucine-, and vitamin D-enriched supplement (21 g protein; 10×/week) or an isocaloric control with RT 3×/week for 13 weeks	greater appendicular muscle mass in the intervention than control groups
Engelen et al. [88]	8 COPD patients (68.1 ± 2.2 y) and 8 healthy subjects (63.1 ± 2.8 y)	29.5g WP or sodium caseinate with a cycle test for 2 days	higher prandial and whole body protein anabolism in CA than WP in COPD patients
Björkman et al. [89]	47 older polymyalgia rheumatica patients (69.5 y)	The experimental group (whey: CA = 80:20) or control group (whey: CA = 20:80) twice daily after RT for 8 weeks	lower limb muscle mass↑, walking speed↑ and chair stand test performance↑ after the post-exercise supplementation
Bemben et al. [101]	42 male subjects (48-72 y)	3 days per week for 14 weeks of RT supplemented with 5g creatine and/or 35g WP	MS↑ and lean body mass↑ after RT with no additional benefits from supplement
Yang et al. [108]	37 elderly men (71.0 ± 4.0 y)	0, 10, 20 or 40g WP isolate after a bout of unilateral leg-based RT.	Greatest MPS in 20g WP at rest; MPS↑ at all protein doses after RT, but greatest MPS in 40g WP.
DeNysschen et al. [109]	28 overweight male subjects (38 y)	3-day-a-week cycle for 12 weeks with 25.8g/day soy versus 26.6g/day WP supplementation	FFM↑, body fat (%)↓, waist/hip↓ and total serum cholesterol↓ in all groups without differences
Reitelseder et al. [125]	17 healthy male subjects (27.0 ± 2.0 y)	whey, CA (0.3g/kg lean body mass), or a noncaloric control drink immediately after heavy RT	MPS↑ at 1-6 h in whey and CA after exercise; phosphorylation of Akt and S6K1↑ after exercise and protein intake; higher 4E-BP1 after whey than CA
Dideriksen et al. [126]	24 elderly persons (68.0 ± 1.0 years)	caseinate intake 30 mins before heavy RT; whey, caseinate or a non-caloric control drink after heavy RT	FSR and MPS does not differ with whey and caseinate after RT, and MPS is similar with caseinate before and after RT.
Human (WP only)			
Katsanos et al. [25]	15 elderly persons (60-85 y)	15g WP, 6.72g EAA, or 7.57g of nonessential amino acids	greater MPS in WP ingestion than ingestion of its constituent EAA content.
Zhu et al. [73]	196 postmenopausal women (74.3 ± 2.7 y)	30g WP or 2.1g protein (placebo) daily for 2 years	no influence on muscle mass or physical function
Kramer et al. [78]	45 healthy older men (69.0 ± 1.0 y)	21g leucine-enriched WP with and without 9g CHO and 3g fat, or an isocaloric mixture containing CHO and fat only	MPS rates↑ after WP intake rather than CHO and fat
Bauer et al. [84]	380 sarcopenic older adults (≥ 65 years)	800 IU vitamin D and 20g leucine-enriched WP (3g leucine) supplement or an iso-caloric control product twice daily for 13 weeks	muscle mass↑ and lower-extremity function↑ after vitamin D and leucine-enriched WP supplement
Hector et al. [86]	40 healthy adults (35-65 y)	27g whey, 26g soy, or 25g CHO twice daily for 12 days	greater MPS in whey than soy or CHO; postprandial MPS↓ less in whey than in soy and CHO
Paddon-Jones et al. [128]	15 healthy elderly individuals (65-79 y)	15g EAAs or WP isolate	net phenylalanine uptake↑ and FSR↑ in both groups, but greatest increase in EAA group
Human (RT only)			
Reeves et al. [100]	18 elder persons (70.7 y)	Leg-extension and leg-press exercises (2 sets of 10 repetitions at 80% of the 5 repetitions maximum) were performed three times weekly for 14 weeks	vastus lateralis muscle fascicle force↑ and muscle volume↑ after exercise
Devries et al. [102]	30 healthy older men (70.0 ± 1.0 y)	14 days step-reduction (SR) (< 1500 steps/day) with 5g citrulline or glycine daily combined with a unilateral low-load RT thrice weekly	FSR↓ in the SR leg; FFM↑ and FSR↑ in the SR + RT leg; no effect of citrulline on muscle
Van Roie et al. [103]	56 older adults (68.0 ± 5.0 y)	3 times weekly for 12 weeks of high- and low-load RT (the bilateral leg press, leg extension and seated row)	no effects of high- and low-load RT on muscle volume, MS and functional capacity
Animals (WP combined with RT)			
Mosoni et al. [27]	healthy male Wistar rats	CA (12%), WP (12%) or WP (18%) with/without polyphenols/antioxidants for 6 months	slower loss of lean body mass in WP (~18%); protein type and polyphenol/antioxidant supplementation had no effects
Butteiger et al. [74]	healthy male Sprague-Dawley rats	20% WP, 20% SP isolate, and two blends (Blend 1 and Blend 2) consisting of ratios of 50:25:25 and 25:50:25 for whey: caseinate: soy, respectively	MPS↑ in all groups; higher FSR peak in Blend 2 than WP at 135 minutes
Kanda et al. [75]	male Sprague-Dawley rats	WP, caseinate, milk protein, or SP (2.4 mL/100 g bw, 3.1g protein/kg bw) immediately after swimming for 2 hours	the fastest initial peak time in MPS after ingestion of WP at different times
Anthony et al. [110]	treadmill-acclimated rats	CHO only, CHO plus SP (CS), or CHO plus WP (CW) immediately after RT	greater phosphorylation of S6K1 and mTOR in CW than in CS
Kanda et al. [127]	male Sprague-Dawley rats	iso-caloric (1100 kJ/100 ml) CHO, CHO plus an amino acid mixture or CHO plus WP hydrolysates immediately after exercise	greater phosphorylation of mTOR, 4E-BP1and S6K1, and FSR in WP compared with amino acid
Animals (RT only)			
Pasini et al. [105]	aged (14-16-month-old) male Wistar rats	a treadmill for 3 or 5 days/week for 8 weeks and compared with age-matched sedentary controls	muscle weight↑, sarcomere volume↑ after 5 days/week treadmill without affecting body weight; substantial impairments in muscle anabolic pathways↓ after exercise

WP: whey protein; CA: casein; CHO: carbohydrate; SP: soy protein; RT: resistance training; MPS: muscle protein synthesis; MS: muscle strength; EAA: essential amino acid; FSR: muscle fractional synthetic rates; S6K1: 70kD ribosomal protein S6 kinase; mTOR: mammalian target of rapamycin kinase; HIIT: high-intensity interval training; BCAA: branch chain amino acid; FFM: fat free mass; 4E-BP1: eukaryotic initiation factor 4E binding protein 1

Taken all together, skeletal muscle and the development of sarcopenia are influenced by gut microbiota, which in turn is affected by WP or RT (WP or RT---gut microbiota---skeletal muscle and sarcopenia). Therefore, we proposed that gut microbiota may be a key factor for WP and/or RT against age-related sarcopenia.

## Conclusion and prospects

In the present review (Fig. 3), we summarized the available evidences regarding the effects of WP and/or RT on age-related sarcopenia in elder people (Table 1). In addition, we also discussed gender differences in skeletal muscle and age-related sarcopenia, and the role of sex hormones in gender differences. It seems that sex hormones could be a potential contributor to muscle health. Furthermore, we proposed that gut microbiota may be a key factor for WP and/or RT against age-related sarcopenia, based on “WP or RT---gut microbiota---skeletal muscle and sarcopenia” pathway.

Appropriate interventions targeting sex hormones and gut microbiota may do great help for preventing and treating age-related sarcopenia according to what we discussed all above. However, there is still no available researches about the direct roles of sex hormones and gut microbiota in age-related sarcopenia. Hence, future studies about these two aspects in age-related sarcopenia are in urgent need.
